# Acute Lymphoblastic Leukemia Presenting Initially as Spinal Cord Compression: When Chemotherapy Alone Is Enough

**DOI:** 10.1155/2020/8735724

**Published:** 2020-03-26

**Authors:** Albert Jang, Kallie N. Kram, Scott N. Berger, Mahmoud R. Gaballa, Lee B. Lu, David R. Dunaway, Courtney N. Miller-Chism

**Affiliations:** ^1^School of Medicine, Baylor College of Medicine, Houston, TX, USA; ^2^Section of Hematology-Oncology, Department of Medicine, Baylor College of Medicine, Houston, TX, USA; ^3^Department of Medicine, Baylor College of Medicine, Houston, TX, USA; ^4^Department of Radiology, Baylor College of Medicine, Houston, TX, USA

## Abstract

Spinal cord compression (SCC) is a rare initial presentation and complication of acute lymphoblastic leukemia (ALL) with nearly all reported cases occurring in the pediatric population. We report a 38-year-old previously healthy man who presented with acute on chronic lower back pain, gait instability, urinary retention, and severe thrombocytopenia. Radiologic examination revealed two soft tissue masses of the thoracic spine associated with compression fractures causing spinal canal narrowing and cord compression. Bone marrow biopsy confirmed the diagnosis of ALL. Immediate initiation of high-dose corticosteroids and systemic chemotherapy resolved the patient's symptoms without radiation therapy or surgical intervention. After two courses of chemotherapy, the patient achieved complete remission in the bone marrow. Rapid administration of chemotherapy alone in this case resulted in a complete resolution of SCC. Given the rarity of this complication in adults, no standardized treatment has been established. The success of this case recommends chemotherapy as the initial management of SCC in chemotherapy-naïve ALL.

## 1. Introduction

Acute lymphoblastic leukemia (ALL) is a malignant neoplasm of early lymphoid precursors. Its incidence is bimodal, with the first peak occurring in childhood (80% of ALL cases) and a second peak around age 50 [1]. ALL has a nonspecific clinical presentation, including B symptoms (fevers, chills, night sweats, and unexpected weight loss), infection, easy bruising, and fatigue. Extramedullary presentations include liver, spleen, and central nervous system (CNS) involvement. It has been reported that 5–10% of patients presented initially with CNS involvement [2–5]. CNS findings can range from headaches, seizures, gait disturbances, focal weakness, and bowel and bladder dysfunction. We report a rare case of an adult with Philadelphia negative B-cell ALL who presented with thoracic vertebral compression fractures and spinal cord compression (SCC) due to epidural masses. Typically, in cancer-associated SCC, radiation or surgical decompression is the initial urgent management; however, rapid initiation of chemotherapy alone is sufficient in the resolution of SCC in highly chemosensitive cancers, such as ALL.

## 2. Case Presentation

**Figure 1:**
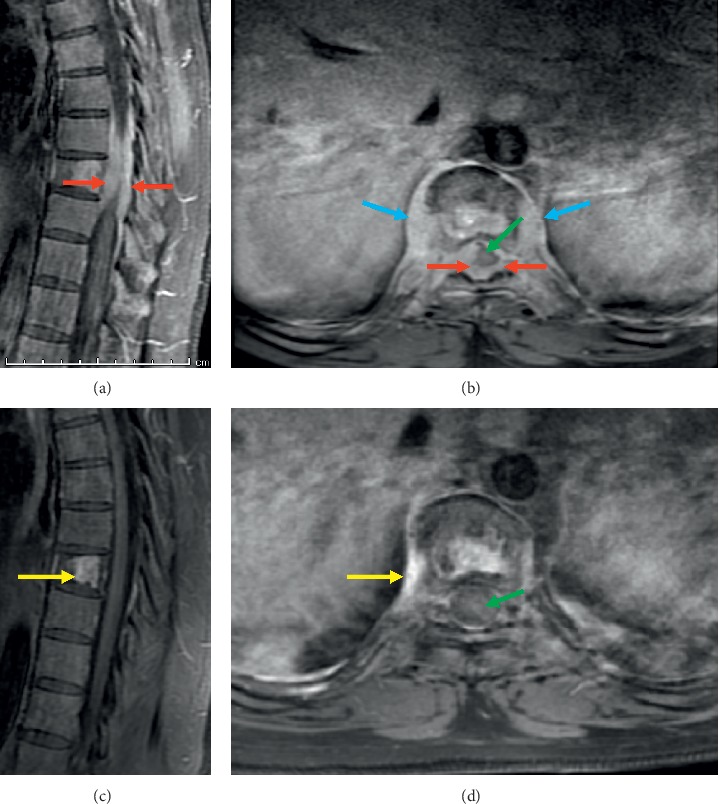
Prevertebral soft tissue mass (blue arrows) and epidural mass (red arrows) as seen on the sagittal (a) and axial (b) postcontrast T1 sequences. Masses have resolved following treatment as seen on the posttreatment sagittal (c) and axial (d) postcontrast T1 sequences. The spinal cord (green arrow), previously encased by tumor, is no longer encased. Only nonspecific reactive changes with enhancement in the T9 vertebrae and prevertebral soft tissues (yellow arrows) remain following treatment.

A 38-year-old previously healthy man presented with acute on chronic nonradiating pain in the thoracic spine. His symptoms worsened four days prior to admission when he began experiencing gait instability, decreased sensation of the buttocks, paresthesia of the inner thighs, lower extremity weakness, and urinary retention. He also noticed occasional bleeding when brushing his teeth over the last few weeks. On admission, he was afebrile with stable vital signs. Physical exam revealed point tenderness with associated paraspinal tenderness in the lower thoracic spine. Neurological exam was pertinent for 5/5 motor strength in all lower extremity muscle groups except left hip flexion of 4/5, hyperreflexia in both knees with positive clonus in the left knee, negative Babinski bilaterally, intact fine touch sensation in the lower extremities, and normal rectal tone. Laboratory findings included hemoglobin of 10.7 g/dL, white blood cell count of 9.1 × 10^3^/*μ*L, and platelet count of 13 × 10^3^/*μ*L. A peripheral smear showed innumerable blasts. Lactate dehydrogenase was 4179 IU/L, and uric acid was 9.9 mg/dL. Magnetic resonance imaging (MRI) of the thoracic spine and computed tomography (CT) of the chest, abdomen, and pelvis both showed compression fractures of the T2 and T9 vertebral bodies. The MRI revealed a 3.0 cm enhancing soft tissue mass that encased and infiltrated the T2 vertebral body and bilateral pedicles and extended through the bilateral T2-T3 neural foramina into the ventral epidural space which resulted in mild canal narrowing. In addition, a 5.0 cm mass encased and infiltrated the T9 vertebral body and extended through the T9-T10 neural foramina and into the epidural space from the level of T7-T8 to the top of T10. Epidural tumor encased the thecal sac and resulted in severe spinal canal narrowing (Figure 1).

Flow cytometry of the peripheral blood and a bone marrow aspirate revealed lymphoblasts positive for CD19, CD20, and terminal deoxynucleotidyl transferase (Tdt), supporting the diagnosis of ALL. Bone marrow biopsy showed >95% blasts. Cytogenetics and fluorescence in situ hybridization (FISH) panel for ALL were normal; Philadelphia chromosome was not present. There was no B-lymphoblastic population detected in the CSF fluid analysis, albeit CSF sampling occurred after chemotherapy was initiated.

Corticosteroids were initiated with immediate improvement of neurologic symptoms within 24 hours. Neurosurgery and radiation oncology were initially consulted. Surgical decompression and radiotherapy were deferred given suspected diagnosis of leukemia. The patient was immediately started on high-dose methotrexate, cytarabine, and rituximab as part of the R-Hyper-CVAD (rituximab with hyperfractionated cyclophosphamide, vincristine, doxorubicin, and dexamethasone) regimen, as well as intrathecal methotrexate. After one course of chemotherapy, his neurological symptoms improved, with resolution of saddle anesthesia, normal patellar reflexes, regular bladder/bowel control, and increased strength with ambulation. After two courses of R-Hyper-CVAD, the patient achieved complete remission in the bone marrow, and subsequent MRI of the spine showed near resolution of the mass at T9 and resolution of previous spinal canal stenosis.

## 3. Discussion

While CNS involvement in patients with ALL is common, there have been only a few documented reports of previously healthy adults presenting with SCC due to extramedullary leukemia in the spinal column at initial diagnosis of ALL. Astute recognition of acute leukemia as a cause of SCC is critical as the management may differ from the typical approach to SCC in patients with solid tumor malignancies.

There are only three cases reported in the literature of a previously healthy adult presenting with SCC in association with ALL [6–8]. The cases are summarized in Table 1. Including our case, all four patients received chemotherapy, two received radiotherapy, and none received surgical intervention. All four patients had rapid responses, with marked improvement in motor strength of the lower extremities.

There is no standardized management of patients presenting with SCC due to ALL. For SCC associated with solid tumors, rapid initiation of steroids to decrease inflammation and to prevent worsening neurological symptoms provides better outcomes [9]. After an initial bolus of steroids, surgery and/or radiotherapy are considered based on the following factors: degree of neurological deficits and radiological involvement, radiosensitivity of the primary cancer, spinal stability, and patient's life expectancy [10]. Patients with SCC from solid tumor metastases managed with both neurosurgery and postoperative radiotherapy had significantly increased ambulation after full treatment compared to patients who received radiotherapy alone [11]. Systemic chemotherapy is routinely not recommended in the initial management of SCC associated with solid tumors due to slower treatment effect on the compressing tumor site compared to surgery and radiation, which are able to deliver rapid local relief to the spinal cord and alleviate further neurological insult. Moreover, most chemotherapy agents utilized for the treatment of solid tumors do not have adequate CNS penetration.

The exception to this general approach occurs when SCC develops in patients with highly chemosensitive tumors. ALL is a very chemosensitive malignancy. Other chemoresponsive malignancies include lymphomas and germ cell tumors, as well as high grade neuroendocrine tumors such as small cell lung cancer [12]. When chemosensitive tumors occur in the spinal cord, the choice of chemotherapy to penetrate the blood brain barrier is important. Compounds including methotrexate, cytarabine, thiotepa, ifosfamide, and temozolomide all have the ability to penetrate the blood brain barrier, and these agents are commonly used in chemotherapy regimens [13]. In fact, the various induction and consolidation regimens for ALL include high-doses of systemic methotrexate and cytarabine due to the propensity of ALL to involve the CNS system. High-dose systemic cytarabine and methotrexate combined with intrathecal chemotherapy are efficacious in the treatment of CNS leukemia [14, 15]. Therefore, rapid initiation of systemic chemotherapy, without surgery or radiation, is an acceptable and perhaps more effective approach to a chemotherapy-naïve patient with SCC secondary to ALL. The advantage of deferring surgical decompression or radiation for SCC in the setting of untreated ALL is the ability to effectively treat the local and systemic disease concurrently. Complications associated with untreated ALL include infections, bleeding, tumor lysis syndrome, and organ failure, particularly if there is leukemic infiltration of the organs. Surgery and radiation will delay the administration of systemic chemotherapy, as chemotherapy may impact wound healing following surgery, and concurrent chemotherapy and radiation may increase myelosuppression. If spinal irradiation is initiated, intrathecal or systemic chemotherapy cannot be initiated within 48–72 hours of radiation [16]. With surgical intervention and radiation, a minimum two-week delay in systemic treatment (due to the radiation normally delivered over ten days) could result in the aforementioned complications and could further delay or alter the systemic treatment plan which may ultimately cure the underlying malignancy. If a patient develops worsening neurologic symptoms following initiation of steroids and/or chemotherapy, or in the relapsed setting, urgent radiation and/or surgery may be appropriate. Consolidative radiation to the spine should also be considered upon completion of induction chemotherapy.

Our patient received neither surgery nor radiotherapy, and high-dose dexamethasone followed by expeditious initiation of R-Hyper-CVAD quickly relieved neurological symptoms and increased motor strength of the lower extremities. High-dose methotrexate and cytarabine, routinely administered during the even courses of the R-Hyper-CVAD protocol, were initiated first in this patient with CNS symptoms because of effective penetration of the blood brain barrier with these agents. Complete remission was achieved in the bone marrow after two courses of R-Hyper-CVAD, and repeat spine imaging obtained after four courses demonstrated nearly complete resolution of the T9 mass without cord compression (Figure 1). The patient has completed eight courses of chemotherapy and remains in complete remission. He has achieved full neurologic recovery and has nearly completed one year of POMP (mercaptopurine, vincristine, methotrexate, and prednisone) maintenance chemotherapy at the time of this submission, including intrathecal prophylaxis every three months. Consolidative radiation to the spine was considered, but this was deferred following negative findings on repeat spinal imaging obtained upon completion of R-Hyper-CVAD.

## 4. Conclusion

Although rare, back pain and SCC could be the initial manifestation of ALL in an adult, particularly in the setting of blood dyscrasia. Malignant SCC is an emergent presentation that often requires rapid management to prevent further neurologic deficits and improve the chance for neurologic recovery. Steroids, followed by surgical decompression and/or radiation therapy, are most often utilized in this setting, particularly when SCC is associated with solid malignancies. However, in the setting of highly chemosensitive cancers (i.e., hematologic malignancies and germ cell tumors), rapid initiation of steroids and systemic chemotherapy offers the advantage of treating both systemic and local disease in a potentially curable cancer, obviating the need for surgery or radiation in the acute setting.

## Figures and Tables

**Table 1:** 

Authors	Year	Age and gender	Initial neurological symptoms	Initial imaging findings	Treatment	Response
Papaliodis et al. [6]	2013	62 y.o. F	Mild weakness with wrist extension and finger extension on the left (strength 4/5); brisk reflexes; positive Hoffmann's sign	Prevertebral lesion from C7 through T2	Radiation and chemotherapy	Rapid resolution of neurological symptoms
Verma et al. [7]	2014	27 y.o. F	Acute onset weakness and numbness of both lower limbs 5 days before coming to the hospital	Lesion measuring 80.6 × 9.3 mm noted in the spinal canal in the dorsal subarachnoid space at T4 through T8 levels compressing the cord	GMALL (German multicenter ALL) protocol with triple intrathecal chemotherapy	Weakness improved within 48 hours
Soliman et al. [8]	2017	29 y.o. M	Acute onset paraplegia with loss of sensation up to T10	Posterior intraspinal epidural mass lesion from T2 through T8	2 weeks of radiotherapy, then Hyper-CVAD	After one course, there was remission, and lower limb strength reached 4/5

## Abbreviations

ALL: Acute lymphoblastic leukemia
CNS: Central nervous system
SCC: Spinal cord compression.
